# Evaluating a Customized Version of ChatGPT for Systematic Review Data Extraction in Health Research: Development and Usability Study

**DOI:** 10.2196/68666

**Published:** 2025-08-11

**Authors:** Jayden Sercombe, Zachary Bryant, Jack Wilson

**Affiliations:** 1 The Matilda Centre for Research in Mental Health and Substance Use, University of Sydney, Jane Foss Russell Building (G02), Level 6, Sydney, 2006, Australia, 612 8627 9380

**Keywords:** artificial intelligence, systematic reviews, data extraction, LLM, ChatGPT, AI, large language models

## Abstract

**Background:**

Systematic reviews are essential for synthesizing research in health sciences; however, they are resource-intensive and prone to human error. The data extraction phase, in which key details of studies are identified and recorded in a systematic manner, may benefit from the application of automation processes. Recent advancements in artificial intelligence, specifically in large language models (LLMs) such as ChatGPT, may streamline this process.

**Objective:**

This study aimed to develop and evaluate a custom Generative Pre-Training Transformer (GPT), named *Systematic Review Extractor Pro*, for automating the data extraction phase of systematic reviews in health research.

**Methods:**

OpenAI’s GPT Builder was used to create a GPT tailored to extract information from academic manuscripts. The Role, Instruction, Steps, End goal, and Narrowing (RISEN) framework was used to inform prompt engineering for the GPT. A sample of 20 studies from two distinct systematic reviews was used to evaluate the GPT’s performance in extraction. Agreement rates between the GPT outputs and human reviewers were calculated for each study subsection.

**Results:**

The mean time for human data extraction was 36 minutes per study, compared to 26.6 seconds for GPT generation, followed by 13 minutes of human review. The GPT demonstrated high overall agreement rates with human reviewers, achieving 91.45% for review 1 and 89.31% for review 2. It was particularly accurate in extracting study characteristics (review 1: 95.25%; review 2: 90.83%) and participant characteristics (review 1: 95.03%; review 2: 90.00%), with lower performance observed in more complex areas such as methodological characteristics (87.07%) and statistical results (77.50%). The GPT correctly extracted data in 14 instances (3.25% in review 1) and four instances (1.16% in review 2) when the human reviewer was incorrect.

**Conclusions:**

The custom GPT significantly reduced extraction time and shows evidence that it can extract data with high accuracy, particularly for participant and study characteristics. This tool may offer a viable option for researchers seeking to reduce resource demands during the extraction phase, although more research is needed to evaluate test-retest reliability, performance across broader review types, and accuracy in extracting statistical data. The tool developed in the current study has been made open access.

## Introduction

The application of artificial intelligence (AI) in health research has the potential to innovate and optimize various research tasks [1]. Systematic reviews are now increasingly conducted as a gold-standard method for evaluating and synthesizing the evidence base [2]. However, this research methodology often takes up significant time, and therefore can be costly, and is subject to human error [3-5]. As large language models (LLMs) and their capabilities rapidly improve, they are now being used to assist with systematic reviews, streamlining a significant portion of health research methodology.

There has been a dramatic rise in the production of systematic reviews, as demonstrated by a 20-fold increase over the past 20-years, equivalent to 80 publications per day [6,7]. Although they have become a valuable resource for informing policy and clinical practice [8], systematic reviews require significant labor and financial costs. A recent analysis concluded that the mean estimated time to complete and publish a systematic review was 67.3 weeks, with an average of five authors per published text [3]. In addition to software and dissemination fees, organizations can expect to pay around USD $140,000 per review [4]. There is further concern for the potential of human error during the screening and extractions stages [5].

In response to this significant burden, there have been strong calls for the use of AI in systematic reviews, making the process more efficient [9]. The oldest applications have focused on the automation of title and abstract screening. Machine learning classifiers, such as support vector machines or complement naive Bayes have been trained to replicate human inclusion/exclusion decisions based on label training data [10,11]. Active learning methods, such as those implemented in softwares such as Covidence [12] or ASReview [13], prioritize the most relevant citations for initial screening. However, these traditional methods still require human involvement to make final decisions on study inclusion, and they do not automate other stages of the review (eg, extraction). Rule-based and natural language processing (NLP)–driven named entity recognition approaches have been used for structured data extraction from full-text articles [14]. Recent developments in deep learning, particularly transformer-based models such as BERT and SciBERT, have improved the performance of such tasks by capturing more nuanced linguistic features [15-17]. The recent advancement in NLP have further contributed toward the development of much-needed extraction tools [18-20]. While these pilot programs demonstrated strong performance, they were largely designed for the extraction of clinical trial data, limiting their application across study designs. Recent focus has shifted toward the popular platform, OpenAI’s ChatGPT [21]. This accessible LLM software is proficient at understanding and processing human language with high speed and accuracy. As the tool can effectively interpret information from complex texts, it may have strong potential for application in the extraction of systematic reviews. In addition, OpenAI recently released the functionality to build customizable versions of ChatGPT. Users can create a Generative Pre-training Transformer (GPT) and tailor it for certain tasks by providing it instructions and assumed knowledge [22]. These applications can be saved and accessed by other users. With the recent GPT functionality, health researchers can now create and share tools specifically designed to carry out systematic review data extraction.

Despite recent advances in AI technology, only three studies to date have reported using LLMs for data extraction. One study briefly reported on the feasibility of ChatGPT for data extraction [23], while others evaluated the performance of ChatGPT-4 [24] and ChatGPT-3.5 [25] ; these studies found moderate performance for extracting complex information but high accuracy for simpler extraction fields [24,25]. While these studies are the first to evaluate the performance of ChatGPT as an extraction tool, further efforts are needed to improve the availability of these programs.

We present a custom GPT program tailored for systematic review extraction, made available as open access. In the current study, we aim to provide a pilot evaluation of the extraction tool, using data from two systematic reviews. Performance of the custom GPT will be compared to that of a human extractor, examining rates of agreement and time taken to extract data.

## Methods

### GPT Building

**Figure 1.**
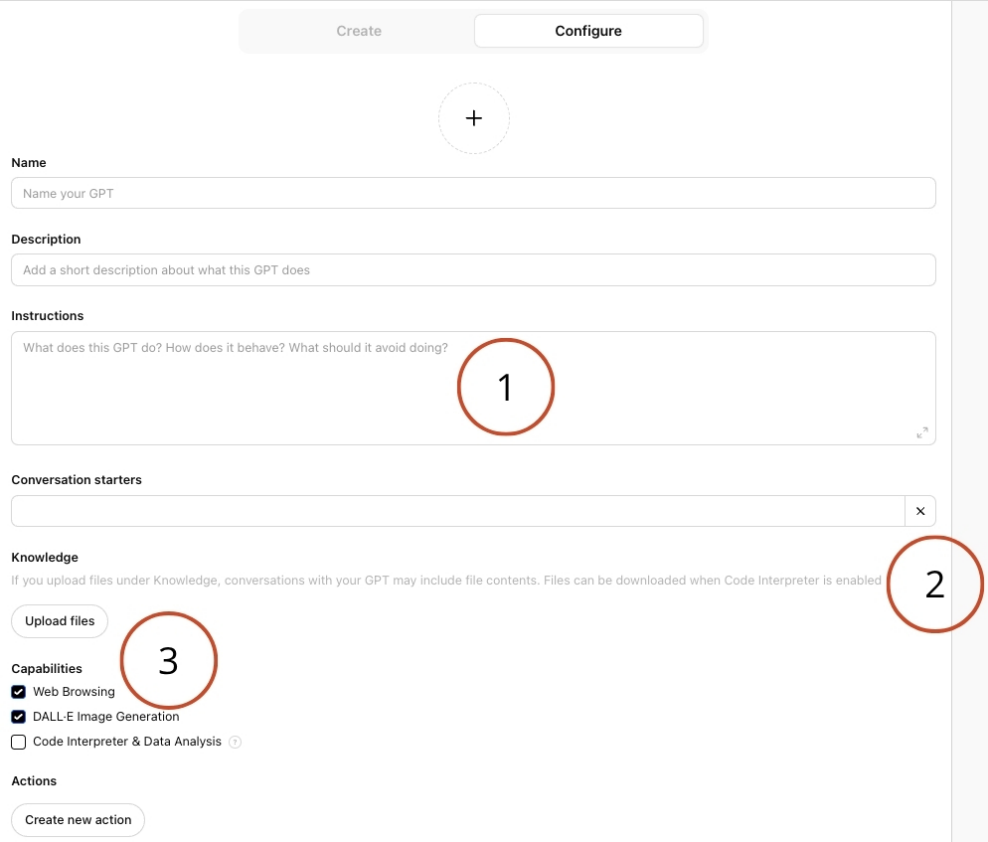
The OpenAI custom GPT Builder interface used to develop and test automated data extraction (1–Instructions, 2–Knowledge, and 3–Capabilities).

OpenAI’s GPT (version 3.5) Builder allows users to create custom versions of ChatGPT that perform specific tasks by combining instructions, knowledge, and capabilities. The GPT builder interface was used to develop a specialized GPT to extract information from academic manuscripts to assist with the extraction phase of two systematic reviews: a methodological review and a systematic review of interventions. The developed GPT is named *Systematic Review Extractor Pro (*Figure 1*)*.

### Prompt Engineering

Detailed prompt engineering guidelines were followed under the Role, Instruction, Steps, End goal, and Narrowing (RISEN) framework [22] to manually configure the GPT’s base context (purpose) and stepped actions to improve its performance in completing the required tasks. Each term refers to specific prompt components that improve the output quality. *Role* provides an identity to the GPT dictating the manner in which it acts, that is,“As an expert in the conduct of systematic reviews you are to....” *Instructions* inform the GPT what task it is to perform, while *Steps* provide a hierarchy of instructions that the GPT is to follow to perform the desired task. *End goal* informs the GPT on the format of desired output and content. Finally, *Narrowing* provides constraints to the GPT including key requirements for performing the given task (eg, setting a word limit on the GPT’s response output).

### Iterative Development of the Tool

The RISEN prompt used to instruct the GPT was iteratively fine-tuned to improve its performance by working through five iterations of the detailed instructions. Differences between iterations were generally focused on providing greater specificity to the prompt and detailed steps to carry out the extraction. Any vagueness in the *Steps* would lead to diverse response ranges for extraction variables, and therefore required the greatest number of changes and troubleshooting. Any changes made to the prompt were systematically tested on dummy health research manuscripts to optimize performance. Due to higher inaccuracy in early stages of extracting statistical results in the template, OpenAI’s user guidelines were followed, to iteratively improve the tool [22]. These guidelines suggest ‘splitting’ or ‘chunking’ complex tasks into smaller ones.

### Reviews for Extraction

A random sample of 10 studies from each of the two systematic reviews (total of 20 studies) were selected to evaluate the performance of the GPT. Review 1 is a systematic review of ecological momentary assessment (EMA) research methodologies for measuring substance use and associated behaviors. Full details are provided in the review protocol which was preregistered on the International Prospective Register of Systematic Reviews (PROSPERO: CRD42023400418). Of specific importance to this review was the extraction of methodological characteristics relating to ecological momentary assessment techniques. Review 2 is a systematic review of the effectiveness of wellbeing interventions for helping professionals. For comprehensive details, refer to the review protocol preregistered on PROSPERO (CRD42023422224). As a review assessing intervention effectiveness, this study included the extraction of statistical results.

### Agreement

Total proportion of agreement was calculated by summing the number of instances where the GPT and human reviewer agreed on extracted data, divided by the total number of applicable fields. Agreement was computed for each study according to subsection, which included study characteristics, participant characteristics, methodological characteristics (only relevant for review 1), and statistical results (only relevant for review 2). See MultimediaAppendices 1  2 for the full list of variables in each subsection. A brief qualitative synthesis of the nature of errors was also conducted.

### Timing

The time taken to extract data for each study was recorded for both the GPT and human reviewer. To enhance practicality, we also recorded the time taken for a human to review the GPT output.

### Ethical Considerations 

This study is a methodological paper which conducts secondary analysis on deidentified and aggregated data and as such, is exempt from requiring ethical approval as per the Australian National Statement on Ethical Conduct in Human Research 2023 [26].

## Results

### Time Taken

The mean extraction time for the human reviewers was 36 minutes. For GPT, the mean time for extraction was 26.6 seconds, and an additional 13 minutes for human review.

### Agreement

For the completed extraction templates for the GPT and human reviewers, see Multimedia Appendix 1 (review 1) and Multimedia Appendix 2 (review 2). As seen in Table 1, overall agreement between the GPT and human reviewer across the 10 studies in review 1 was 91.45%; agreement was the highest for study (95.25%) and participant characteristics (95.03%) and the lowest for methodological characteristics (87.07%). For the 10 studies in review 2, overall agreement was 89.31%, similar to review 1. The highest agreement was observed in study (90.83%) and participant (90.00%) characteristics. Lower agreement was recorded in extraction fields relating to statistical results (77.50%).

**Table 1.** Agreement between human reviewers and GPT for data extraction by manuscript subsections.

Manuscript subsection	Review 1 (agreement %)	Review 2 (agreement %)
Study characteristics	95.25	90.83
Participant characteristics	95.03	90.00
Methodological characteristics	87.07	—^a^
Results	—^a^	77.50
Overall agreement	91.45	89.31

a Data not applicable for review.

### Qualitative Summary of Errors

Across both Review 1 and 2, the most common error made by the GPT was reporting an extraction field as “not specified,” despite the relevant information being present in the study manuscript (see MultimediaAppendices 1  2). These types of errors accounted for a high proportion of the inaccuracies, particularly where only p-values were sometimes reported rather than the required measures of effect.

While there were instances of errors resembling hallucination (eg, describing a randomized controlled trial as a pre-post study), these were few in number. When the GPT erroneously inserted information, it typically mistakenly drew data from other parts of the paper, rather than fabricating the information entirely, For example, several errors occurred in sample size extraction, such as reporting the baseline sample as the follow-up sample.

### Instances Where the GPT Was More Accurate That the Human Reviewer

In review 1, there were 14 instances where the GPT correctly extracted fields, and the human reviewer was incorrect (equating to 3.25% of the overall number of fields). In Review 2, the GPT was correct in four fields where the human reviewer was incorrect with a rate of 1.16%.

## Discussion

### Principal Findings

This study evaluated the performance of a custom-configured LLM, specifically a GPT, in automating the data extraction phase of two systematic reviews. This application of AI for extraction provides initial insights into addressing challenges in systematic review methodology, such as time consumption, labor intensity, and potential for human error.

Even when accounting for human review, the custom GPT took less than 14 minutes to complete extraction of each study, 22 minutes faster than human extraction. The 26.6 seconds it took for extraction alone was comparable to a study by Gue et al [25], where ChatGPT-3.5 took approximately 17 seconds per study compared to 77 minutes by a reviewer. As such, the findings of prior research alongside the current study emphasize the processing speed of AI and its potential to significantly reduce the time demands and resultant costs associated with systematic reviews. This efficiency could facilitate more timely summaries of evidence-based practices and support researchers in managing large volumes of literature.

The high overall agreement rates observed in this study between the GPT and human extraction for review 1 (91.45% overall) and 2 (89.31%), suggest that LLMs may have potential to support automation of the extraction of data from academic manuscripts. Consistent across review 1 and 2, the GPT was particularly effective at extracting study (95.25% and 90.83%) and participant characteristics (95.05% and 90%, respectively). This is likely due to the homogeneity in participant and study characteristics that are reported in the extracted manuscripts, reflecting reporting standards upheld by academic journals.

However, agreement rates were lower when extracting methodological characteristics (77.88%) and results (77.50%) sections. Early iterations were improved by ‘splitting’ the task by extracting results separately to the rest of the extraction template. Results are challenging for GPT to extract, as studies used varied methods of analysis and some statistical results were only reported in tables. As LLMs favor extracting data from text information, additional prompting was required to ensure that tables were thoroughly and consistently incorporated into the information extraction sequence.

In this study, a small percentage of instances where the GPT outperformed the human extractor were observed. This demonstrates the potential for AI to identify patterns and insights that may be overlooked in human extraction. Human extractors can be prone to fatigue which may have led to these small inconsistencies. The preliminary findings of this study suggest that LLMs may have utility in identifying errors made by human extractors.

Overall, in accordance with previous research, LLMs excel in quickly extracting simple data (eg, participant demographics [25]. The purpose-built GPT evaluated in the current study was less accurate while extracting more complex fields compared to simple fields (agreement ranged from 77.50% to 95.25%) but appears to be relatively accurate in comparison to extraction using the generic ChatGPT model where agreement rates were as low as 41.2% [25]. A study of 87 human reviewers found error rates of 28.3% to 31.2%, corresponding to agreement rates of 68.8% and 71.7% with a gold-standard reviewer [27]. These rates remained relatively consistent across reviewer experience level and, similar to the findings of the current study, were more accurate for the extraction of participants and study characteristics compared to statistical results. Overall, the GPT evaluated in the current study demonstrated comparable error rates to those reported by human reviewers.

In its current format, researchers may consider the use of this tool as a second extractor, when they lack funding, resourcing, or time for a team member to perform the task (Textbox 1). The overall agreement rate of the custom GPT was approximately 90%, and the GPT had utility in identifying some human errors. Caution should be exercised while using the tool to extract information in areas where it showed lower accuracy, in particular results and methodological characteristics.

Textbox 1. **A guide to using the Systematic Review Extractor Pro for data extraction.**

How to use the tool

1. Log into a ChatGPT account and access the ‘Systematic Review Extractor Pro’ GPT [28].
2. Copy and paste your extraction template variable headings into the GPT message bar (any text format).
3. Upload a PDF file of the study from which you want to extract data.
4. Copy the output to an Excel file. If needed, use the ‘Convert Text to Table’ functionality, separating by ‘|’.

Optimizing use of this tool

- Be explicit with the outcomes in your extraction template. For example, “Mental health outcomes (specify scales and subscales used in bullet point format)” or “modality (in-person; digital; telephone).” For an example, see the authors templates in MultimediaAppendices 1  2.
- With more complex aspects of the template such as statistical results, you may have better accuracy by ‘chunking’ the task. This involves splitting complex tasks into simpler ones. To do this, provide the tool with the results section of the extraction template separate to the rest of the template.

Citing this tool and other considerations

- To reference the use of this tool in publications, please cite this paper.
- Consider the copyright limitations of articles when uploading them to the tool.

### Strengths and Limitations

Building on previous work exploring the capacity of ChatGPT 3, 3.5 and 4 to perform data extraction [23-25], this study tested the performance of a custom-built GPT across two distinct systematic reviews. To the best of our knowledge, this study highlights the first empirical investigation of a custom OpenAI GPT to perform data extraction in a systematic review.

Our study has several limitations. When determining agreement between extractors, there is always a degree of subjectivity between coding any qualitative responses, which can either overestimate or underestimate agreement rates. The current study observed agreement rates across 20 studies. Testing a larger sample size would improve confidence in the tool. In terms of limitations of the tool itself, the tool’s construction on the GPT Builder may pose replicability challenges of the output due to the constantly evolving nature of the LLM. The benefit of this aspect is that the tool will automatically update as ChatGPT releases improved versions of their model [29]. However, future research should evaluate the test-retest validity of this custom GPT.

### Conclusions

This study serves as promising early evidence for the application of a custom-built GPT in conducting systematic review data extraction. The findings of this study are consistent with the growing sentiment suggesting AI can enhance the efficiency of systematic reviews and reduce cost and time while maintaining accuracy [4]. By encouraging open discourse regarding the role of AI in research, researchers can contribute to the development of robust, transparent, and reproducible scientific practice. This is a rapidly advancing field of technology and OpenAI frequently releases new versions of ChatGPT that show improved performance and efficiency [29]. It is likely that with further advancement of AI, LLMs may be relied on as a sole reviewer for data extraction.

## Supplementary material

10.2196/68666 Multimedia Appendix 1 Systematic review 1 - agreement file.

10.2196/68666 Multimedia Appendix 2 Systematic review 2 - agreement file.

## Abbreviations

AI: artificial intelligence
GPT: Generative Pre-Training Transformer
LLM: large language model
RISEN: Role, Instruction, Steps, End goal, and Narrowing

## References

[1] Rajpurkar P, Chen E, Banerjee O, Topol EJ (2022). AI in health and medicine. Nat Med.

[2] Higgins JPT, Thomas J, Chandler J, Cumpston M, Li T, Page MJ, Welch VA (2024). Cochrane Handbook for Systematic Reviews of Interventions Version 65.

[3] Borah R, Brown AW, Capers PL, Kaiser KA (2017). Analysis of the time and workers needed to conduct systematic reviews of medical interventions using data from the PROSPERO registry. BMJ Open.

[4] Michelson M, Reuter K (2019). The significant cost of systematic reviews and meta-analyses: a call for greater involvement of machine learning to assess the promise of clinical trials. Contemp Clin Trials Commun.

[5] Mathes T, Klaßen P, Pieper D (2017). Frequency of data extraction errors and methods to increase data extraction quality: a methodological review. BMC Med Res Methodol.

[6] Hoffmann F, Allers K, Rombey T (2021). Nearly 80 systematic reviews were published each day: observational study on trends in epidemiology and reporting over the years 2000-2019. J Clin Epidemiol.

[7] Johnson BT, Hennessy EA (2019). Systematic reviews and meta-analyses in the health sciences: best practice methods for research syntheses. Soc Sci Med.

[8] Chalmers I, Fox DM (2016). Increasing the incidence and influence of systematic reviews on health policy and practice. Am J Public Health.

[9] van Dijk SHB, Brusse-Keizer MGJ, Bucsán CC, van der Palen J, Doggen CJM, Lenferink A (2023). Artificial intelligence in systematic reviews: promising when appropriately used. BMJ Open.

[10] O’Mara-Eves A, Thomas J, McNaught J, Miwa M, Ananiadou S (2015). Using text mining for study identification in systematic reviews: a systematic review of current approaches. Syst Rev.

[11] Wallace BC, Small K, Brodley CE, Trikalinos TA Active learning for biomedical citation screening.

[12] (2025). Covidence systematic review software.

[13] van de Schoot R, de Bruin J, Schram R (2021). ASReview: Open source software for efficient and transparent active learning for systematic reviews. Int J Digit Curation.

[14] Jonnalagadda SR, Goyal P, Huffman MD (2015). Automating data extraction in systematic reviews: a systematic review. Syst Rev.

[15] Beltagy I, Lo K, Cohan A SciBERT: a pretrained language model for scientific text.

[16] Khalil H, Ameen D, Zarnegar A (2022). Tools to support the automation of systematic reviews: a scoping review. J Clin Epidemiol.

[17] van de Schoot R, de Bruin J, Schram R (2021). An open source machine learning framework for efficient and transparent systematic reviews. Nat Mach Intell.

[18] Golinelli D, Nuzzolese AG, Sanmarchi F (2022). Semi-Automatic systematic literature reviews and information extraction of COVID-19 scientific evidence: description and preliminary results of the COKE project. Information.

[19] Panayi A, Ward K, Benhadji-Schaff A, Ibanez-Lopez AS, Xia A, Barzilay R (2023). Evaluation of a prototype machine learning tool to semi-automate data extraction for systematic literature reviews. Syst Rev.

[20] Zhang T, Yu Y, Mei J, Tang Z, Zhang X, Li S (2020). Unlocking the power of deep PICO extraction: step-wise medical NER identification. arXiv.

[21] (2023). ChatGPT. Open AI.

[22] (2024). Prompt engineering. Open AI.

[23] Mahuli SA, Rai A, Mahuli AV, Kumar A (2023). Application ChatGPT in conducting systematic reviews and meta-analyses. Br Dent J.

[24] Khraisha Q, Put S, Kappenberg J, Warraitch A, Hadfield K (2024). Can large language models replace humans in systematic reviews? Evaluating GPT-4’s efficacy in screening and extracting data from peer-reviewed and grey literature in multiple languages. Res Synth Methods.

[25] Gue CCY, Rahim NDA, Rojas-Carabali W (2024). Evaluating the OpenAI’s GPT-3.5 Turbo’s performance in extracting information from scientific articles on diabetic retinopathy. Syst Rev.

[26] Council and Universities Australia (2023). National Statement on Ethical Conduct in Human Research.

[27] Horton J, Vandermeer B, Hartling L, Tjosvold L, Klassen TP, Buscemi N (2010). Systematic review data extraction: cross-sectional study showed that experience did not increase accuracy. J Clin Epidemiol.

[28] Systematic review extractor pro. ChatGPT.

[29] Achiam J, Adler S, Agarwal S, OpenAI (2024). GPT-4 technical report. arXiv.

